# Bioluminescence Method for *In Vitro* Screening of Plasmodium Transmission-Blocking Compounds

**DOI:** 10.1128/AAC.02699-16

**Published:** 2017-05-24

**Authors:** Raquel Azevedo, Marija Markovic, Marta Machado, Blandine Franke-Fayard, António M. Mendes, Miguel Prudêncio

**Affiliations:** a Instituto de Medicina Molecular, Faculdade de Medicina, Universidade de Lisboa, Lisbon, Portugal; b Leiden Malaria Research Group, Department of Parasitology, Leiden University Medical Center, Leiden, The Netherlands

**Keywords:** Plasmodium berghei, sporogony, mosquito stages, malaria, *in vitro* cultures

## Abstract

The sporogonic stage of the life cycle of Plasmodium spp., the causative agents of malaria, occurs inside the parasite's mosquito vector, where a process of fertilization, meiosis, and mitotic divisions culminates in the generation of large numbers of mammalian-infective sporozoites. Efforts to cultivate Plasmodium mosquito stages *in vitro* have proved challenging and yielded only moderate success. Here, we describe a methodology that simplifies the *in vitro* screening of much-needed transmission-blocking (TB) compounds employing a bioluminescence-based method to monitor the *in vitro* development of sporogonic stages of the rodent malaria parasite Plasmodium berghei. Our proof-of-principle assessment of the *in vitro* TB activity of several commonly used antimalarial compounds identified cycloheximide, thiostrepton, and atovaquone as the most active compounds against the parasite's sporogonic stages. The TB activity of these compounds was further confirmed by *in vivo* studies that validated our newly developed *in vitro* approach to TB compound screening.

## INTRODUCTION

Malaria remains a formidable public health threat, accounting for the deaths of nearly half a million people annually (1). It is caused by apicomplexan parasites of the genus Plasmodium, five species of which, Plasmodium falciparum, P. ovale, P. vivax, P. malariae, and P. knowlesi, are able to infect humans.

The complex life cycle of the Plasmodium parasite starts when an infected female Anopheles mosquito vector bites and injects sporozoites under the skin of a mammalian host (2). Sporozoites enter the bloodstream, reach the liver, and eventually invade a hepatocyte, where they undergo an extensive replication process that culminates in the formation of thousands of merozoites (3). Following the asymptomatic liver stage of infection, released merozoites infect erythrocytes, thus initiating the symptomatic blood stage of infection. A small proportion of parasite blood stages differentiates into female and male gametocytes, which can be taken up by the mosquito vector upon a subsequent blood meal (4). The sporogonic phase of the Plasmodium life cycle occurs inside the mosquito vector. Ingested gametocytes mature and form female and male gametes, a process induced by environmental triggers such as a drop in the temperature of the infected blood, a rise in pH from 7.3 to 7.8, and the presence of gametocyte activation factors such as xanthurenic acid (5). Gamete fertilization results in the formation of a zygote, which then transforms into the motile, banana-shaped ookinete. After 24 h, these parasite forms leave the mosquito midgut, migrate toward the midgut wall, traverse the epithelial cells, and eventually arrest between the midgut epithelium and the basal lamina. Here, ookinetes round up and develop into oocysts, where sporogony, a process of asexual replication driven by mitotic divisions, takes place, leading to the development of numerous sporozoites. After 15 to 21 days in the mosquito vector, the oocyst wall bursts, releasing the sporozoites, which are transported through the hemocoel toward the salivary glands, where they remain until they are transmitted to another mammalian host (6, 7).

The complex life cycle of Plasmodium parasites significantly contributes to the enormous challenge posed by malaria eradication. The compounds most commonly used for treatment and prophylaxis nowadays target the asexual blood stages of infection (8). However, to support eradication of the parasite, combination therapies should address three major issues: transmission of the pathogen, radical cure of P. vivax malaria, and the emergence of compound resistance (9). Thus, transmission-blocking (TB) strategies emerge as a crucial component of a multifaceted antimalarial strategy (10). Such strategies aim at reducing the prevalence of infection in communities where malaria is endemic by targeting Plasmodium's mosquito stages, impairing the transmission of the disease from an infected host undergoing treatment to the vector, and clearing the parasite from the vector (11). In this context, an increased understanding of the invertebrate vector stages of the malaria parasite, as well as effective methods to identify TB compounds, is urgently required.

Efforts to cultivate Plasmodium parasites *in vitro* have been hindered by the complexity of the parasites' hosts, the human host and the arthropod vector, each having its own physiological, metabolic, and nutritional requirements, which are difficult to reproduce *in vitro* (12). Nevertheless, *in vitro* culturing of Plasmodium mosquito stages has been reported for different species of these parasites, including P. gallinaceum (13), P. falciparum (14), P. berghei (15), and P. yoelii (16). Early studies of *in vitro* production of P. berghei ookinetes employed minimum essential medium for cultivation of gametocytes produced *in vivo*. In these studies, 1% or less of the initial macrogametocytes were found to originate ookinetes (17). The *in vitro* production of P. berghei gametocytes and their infectivity toward mosquitoes were first demonstrated by Janse et al., who achieved the transformation of 44% of macrogametocytes into ookinetes (18). Later, transformation of P. berghei zygotes into ookinetes (19) and ookinete transformation into early oocysts (20) were also achieved in the absence of cells. However, studies of P. gallinaceum showed that zygote-to-ookinete transformation rates of up to 75% and substantially increased ookinete longevity were observed in the presence of insect cells (21). P. berghei oocyst-to-sporozoite transformation *in vitro* was first described in 2002 (15). In that study, mature oocysts were obtained in 15 days of culturing employing Matrigel and cocultures with Drosophila cells to mimic the mosquito's basal lamina, which is expected to provide the necessary cues for oocyst transformation (22, 23). Under these conditions, Drosophila cells produce laminin, which has been suggested to coat ookinetes as they pass through the mosquito's midgut epithelium (24), and annexin, which was shown to bind to ookinetes during the invasion of the mosquito midgut (25). Nevertheless, Carter et al. subsequently showed that no basal lamina components are required to trigger P. berghei ookinete-to-oocyst transformation *in vitro* and defined a minimal medium that supports transformation and oocyst growth in the absence of Matrigel or cocultured cells (26). However, maintenance of the parasites in minimal medium did not permit complete sporogonic development, and the duration of oocyst viability was reduced to 7 days (26).

The complexity of currently available methods of *in vitro* cultivation of Plasmodium sporogonic stages renders their reproducibility very challenging. Nevertheless, a suitable *in vitro* culturing system for such stages could represent an important tool for the development of TB interventions, as well as sporozoite-based vaccines, the sole immunization strategies shown to convey sterile immunity to malaria (27). Here, we have optimized an *in vitro* system for culturing P. berghei mosquito stages and screening TB compounds, employing a bioluminescence-based output of increased simplicity. Our results show that this system can be employed for effective and fast screening of TB compounds and for identification of the specific stages of parasite development upon which these compounds act. Finally, this method may contribute to a better understanding of the sporogonic stage of the parasite's life cycle.

## RESULTS

### *In vitro* culturing of P. berghei mosquito stages.

**FIG 1:**
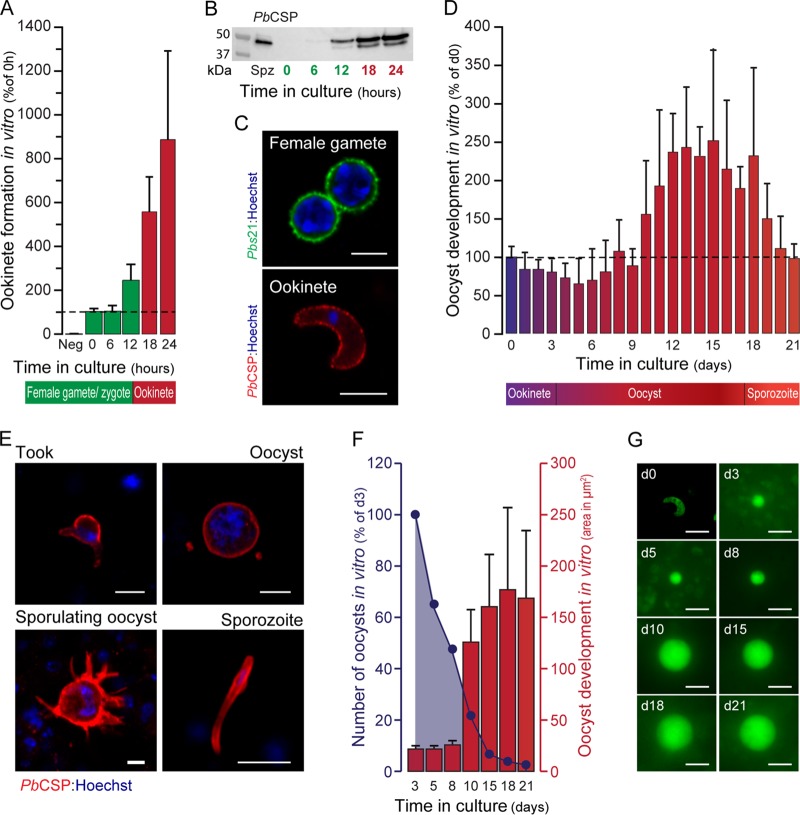
A new system for culturing and detecting P. berghei mosquito stages. (A) Bioluminescence measured in relative luminescence units (RLU) at different time points throughout the formation and maturation of ookinetes, represented as a percentage of the RLU measured at the start of culture, 0 h. Neg corresponds to the uninfected blood sample used as a negative control. Results are expressed as the mean ± the standard deviation. (B) Western blot analysis of *Pb*CSP protein expression throughout the transformation of gametes into ookinetes *in vitro*. Salivary gland sporozoites were used as a positive control. (C) Immunofluorescence microscopy analysis of *Pb*CSPGFP-Luc gametes and ookinetes (green, Pbs21 protein; red, *Pb*CSP; blue, nuclei). Scale bars, 5 μm. (D) Bioluminescence measured in RLU throughout 21 days of oocyst culture, represented as a percentage of the RLU at time zero (d0) of culture. Results are expressed as the mean ± the standard deviation. (E) Representative images of immunofluorescence staining of P. berghei tooks, oocysts, sporulating oocysts, and free sporozoites (red, *Pb*CSP; blue, nuclei). Scale bars, 5 μm. (F) Quantification of oocyst numbers and development by live fluorescence microscopy. Results are expressed as the mean ± the standard deviation. d3, day 3. (G) Immunofluorescence staining of *Pb*CSPGFP-Luc ookinetes (time zero [d0]) and live imaging of *Pb*CSPGFP-Luc oocysts (day 3 [d3] to day 21 [d21]) cultured *in vitro*. Scale bars, 10 μm.

P. berghei mosquito stages were cocultured with Drosophila S2 cells in fetal bovine serum (FBS)-supplemented Schneider's medium for 21 days, up to oocyst development and sporozoite production. A P. berghei transgenic line with a cassette expressing green fluorescent protein (GFP) and luciferase under the control of P. berghei circumsporozoite protein (*Pb*CSP) promoter regions, was employed (*Pb*CSPGFP-Luc; see Fig. S1 in the supplemental material), enabling the assessment of the *in vitro* development of the parasite's mosquito stages by bioluminescence. Blood was collected from *Pb*CSPGFP-Luc-infected mice and washed, and between 3 × 10^5^ and 5 × 10^6^ gametocytes were subsequently cultured as described in Materials and Methods. An ookinete formation rate of 25 to 33% of the total number of female gametocytes/gametes was estimated. Bioluminescence was measured at specific time points thereafter and increased steadily over the initial 24 h of culture, corresponding to the parasite's progress from the gametocyte to the ookinete stage (Fig. 1A). The intensity of the bioluminescence signal is in accordance with an unexpected pattern of expression of *Pb*CSP (Fig. 1B) on ookinetes and positively correlates with ookinete numbers (see Fig. S2B in the supplemental material). Immunofluorescence microscopy analyses showed that expression of *Pb*CSP by ookinetes is a feature of the *in vitro* culturing process and is not an artifact of *Pb*CSPGFP-Luc parasites (Fig. 1C), as it is equally observed in cultures of *Pb*GFP-Luc_Con_, a parasite whose constitutive luciferase expression is not driven by the *Pb*CSP promoter (see Fig. S3A in the supplemental material). Western blot analysis further confirmed that *Pb*CSP expression could be detected after 24 or 48 h of *in vitro* culturing of either *Pb*CSPGFP-Luc or *Pb*GFP-Luc_Con_ (see Fig. S3B in the supplemental material), whereas the protein was not detected in mosquito midgut samples collected 24 and 48 h after mosquito infection with those parasites and analyzed by Western blotting or immunofluorescence microscopy (see Fig. S3B and C in the supplemental material, respectively).

Purified ookinetes (10 to 40% purity) were then cultured for an additional 21 days, with daily bioluminescence measurements (Fig. 1D). A detailed immunofluorescence microscopy analysis of fixed samples further showed the development of transforming ookinete (took) forms, followed by oocysts containing multiple nuclei, with the presence of *Pb*CSP on the parasite's surface, as well as oocyst sporulation and free sporozoites from day 18 onward (Fig. 1E). Our data also showed that oocyst size increased and sporulation started on day 10, reaching a maximum between days 13 and 18, while oocyst numbers decreased as the culturing process progressed (Fig. 1F), from an initial ∼5,000 oocysts on day 3 to ∼1,500 on day 10 and ending with ∼60 by day 21. Live fluorescence microscopy analyses at specific time points showed that ookinetes develop into growing oocysts and that the peak of signal intensity correlates with oocyst enlargement and sporulation (Fig. 1G).

Collectively, our data reveal a culture system that effectively reproduces the mosquito stages of P. berghei parasites *in vitro*, from gametocytes to sporulating oocysts. Crucially, we also show that by employing a luciferase-expressing P. berghei line, the parasite's sporogonic development can be monitored by bioluminescence analysis.

### Effects of antiplasmodial compounds on Plasmodium mosquito stages *in vitro*.

**FIG 2:**
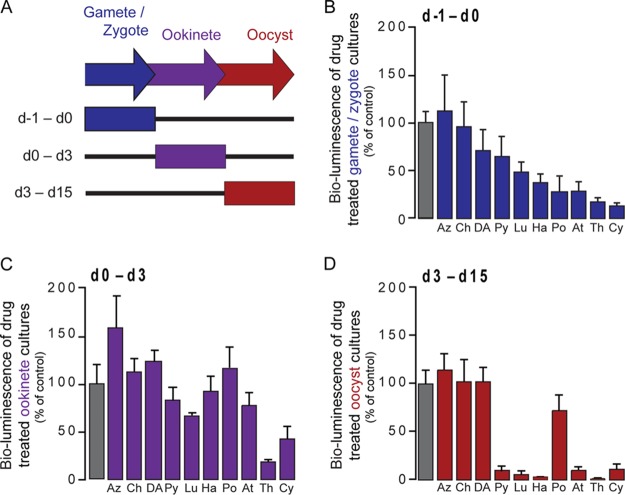
Effects of selected compounds against Plasmodium mosquito stages *in vitro*. (A) Schematics of the progress of the parasite culturing process highlighting the different schedules of compound treatment employed. d, day. (B) Assessment of compound effects on ookinete formation and development, expressed as percentages of inhibition of P. berghei ookinete formation. (C) *In vitro* activities of selected compounds against early stages of oocyst development. (D) *In vitro* activities of selected compounds on young oocysts and subsequent developmental stages. Ten compounds, Az, Ch, DA, Py, Lu, Ha, Po, At, Th, and Cy, were screened at a concentration of 10 μM. Bars correspond to RLU measurements represented as percentages of the RLU of the DMSO control. Results are expressed as the mean ± the standard error of the mean.

The newly developed method described above can be employed to assess the activities of potential TB compounds *in vitro*. To prove the principle of this method, 10 well-established antimalarial compounds (azithromycin [Az], chloroquine [Ch], dihydroartemisinin [DA], pyrimethamine [Py], lumefantrine [Lu], halofantrine [Ha], pyronaridine [Po], atovaquone [At], thiostrepton [Th], and cycloheximide [Cy]) belonging to different classes and with various expected effects on the development of Plasmodium mosquito stages were selected. The compounds' abilities to inhibit different phases of parasite development were assessed by bioluminescence analysis following compound addition at various stages of the culturing process (Fig. 2A).

### Identification of compound effects on ookinete formation and development.

To assess the effects of the selected compounds on ookinete formation and subsequent development, compounds were added to gametes and zygotes (after 1 h of culture incubation). Bioluminescence was measured after 24 h of the culturing process and compared with that of control cultures to which only compound vehicle was added (Fig. 2B). Our results show that At, Po, Th, and Cy have a strong effect on parasite development, whereas Ha and Py have a small effect on the zygote-to-ookinete transition stage and Az, Ch, DA, and Lu have no discernible effect on parasite development.

### Identification of compound effects on oocysts.

To assess the compounds' activities on oocyst formation and early stages of development, the compounds were added to purified ookinetes and parasites were harvested after 3 days of culturing (Fig. 2C). Compound effects on late oocyst development were assessed by adding the compounds to already formed young oocysts (after 3 days of oocyst culturing) and harvesting the parasites after 15 days of culturing (Fig. 2D). The greatest efficacy against early oocyst stages was obtained with Th and Cy, while the strongest effects against later stages of oocyst growth were observed with Th, Cy, Py, At, Ha, and Lu. The half-maximal inhibitory concentrations (IC_50_s) calculated for Cy, Py, and Th upon addition of the compounds to young oocysts were 0.28 ± 0.32, 1.93 ± 1.72, and 1.16 ± 0.10 μM, respectively (see Fig. S4 in the supplemental material).

Collectively, our results indicate that our bioluminescence-based method can be employed for the *in vitro* identification of compounds with activity against Plasmodium mosquito stages. The assay may also serve to distinguish between the different phases of parasite development where the compounds may be acting. Nonetheless, the different incubation times employed in the early- and late-stage assays do not allow exclusion of the possibility that apparent differences in the compounds' stage specificity may also result from their faster or slower mode of action.

### *In vivo* effects of compounds identified in the *in vitro* assay.

**FIG 3:**
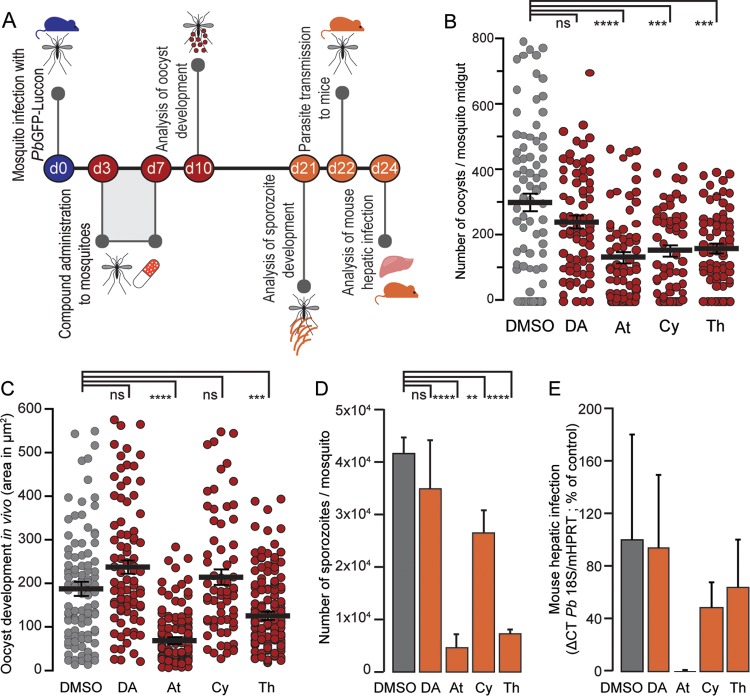
Compound activity on P. berghei sporogonic development in infected mosquitoes and sporozoite infectivity of a mammalian host. (A) Schematics of assessment of *in vivo* compound activity on oocysts and sporozoites and liver parasite loads of mice infected by mosquitoes treated with selected compounds. d, day. (B) *In vivo* activities of selected compounds on the number of oocysts developing in mosquito midguts. Similar population sizes were analyzed, and results are expressed as the mean ± the standard error of the mean. (C) *In vivo* activities of selected compounds on oocyst development in mosquito midguts. Oocyst areas in square micrometers are presented for random samples of parasites observed in midguts of treated mosquitoes. Results are expressed as the mean ± the standard error of the mean. (D) *In vivo* activities of selected compounds on sporozoite formation. Results are expressed as the mean ± the standard deviation. (E) Quantification of *in vivo* hepatic infection of mice infected by treated mosquitoes. Results are expressed as the mean ± the standard error of the mean. Compounds were screened at a concentration of 50 μM. **, *P* < 0.0001; ***, *P* < 0.001; ****, *P* < 0.005; ns, not significant.

To test our newly developed screening method, the TB potential of At, Cy, and Th, which displayed potent activity against the parasite's mosquito stages *in vitro*, was assessed *in vivo*. DA, which displayed negligible activity in our *in vitro* screening, was employed as a negative control in these studies. Anopheles stephensi mosquitoes infected 1 day earlier by feeding on *Pb*GFP-Luc_con_-infected mice were starved for 2 days and subsequently allowed to feed on aqueous solutions containing the selected compounds at 50 μM (Fig. 3A). Ten days later, a fraction of the total number of mosquitoes in each group was dissected, their midguts were collected, and oocyst numbers (Fig. 3B) and development (Fig. 3C) were quantified by microscopy. Infection of the remaining mosquitoes in each group was allowed to proceed for an additional 11 days, after which either (i) mosquitoes were dissected and salivary gland sporozoites were counted (Fig. 3D) or (ii) mosquitoes were allowed to bite naive mice and the parasite loads in the livers of these mice were determined 46 h later by quantitative real-time PCR (qRT-PCR) (Fig. 3E). Our results show that oocyst numbers in mosquitoes that fed on At-, Cy-, and Th-containing solutions were similar and significantly lower than those present in the midguts of vehicle- or DA-treated mosquitoes (Fig. 3B), whereas At and Th also significantly impacted oocyst development (Fig. 3C). Consequently, the number of sporozoites in the salivary glands of mosquitoes treated by any of these drugs is significantly decreased, and more markedly so in the case of treatment with At or Th (Fig. 3D). Importantly, our results also show that administration of the identified TB compounds to infected mosquitoes has a clear impact on an ensuing hepatic infection of naive mice bitten by these mosquitoes (Fig. 3E). Of note, these data further suggest that sporozoite viability is strongly impaired by At and is also impacted by Cy or Th. Collectively, our results indicate that compound activity against sporogonic stages, as identified in our *in vitro* assay, is predictive of the activity of these compounds against parasite development *in vivo* in a previously infected mosquito vector, with a clear impact on transmission. Overall, these data show that our *in vitro* assay can identify compounds active against Plasmodium mosquito stages and inform their selection for further evaluation as potential TB compounds.

## DISCUSSION

In the present study, we showed that the development of the parasite's mosquito stages can be monitored *in vitro* by bioluminescence measurements, enabling a new method to screen potential TB compounds. We employed a modified version of the *in vitro* culturing conditions described by Al-Olayan et al. (15) to obtain sporulating oocysts from gametocyte cultures of a transgenic P. berghei parasite line, *Pb*CSPGFP-Luc, which expresses GFP and luciferase at the mature ookinete, oocyst, and sporozoite stages of its life cycle. Xanthurenic acid was incorporated in the ookinete medium as a promoter of exflagellation, and we established an optimal pH for ookinete transformation of 7.6, in comparison with the pH of 8.4 established by Al-Olayan et al. In the culturing system described here, oocysts were successfully maintained and sporozoites were released by using a culture medium composed solely of Schneider's medium, which is a source of both glutamine and sodium bicarbonate, and FBS, which is composed of several growth factors that might be important for parasite maintenance *in vitro* (28). A pH of 6.6 to 6.8 was employed for oocyst development, whereas in the study of Al-Olayan et al., the optimal oocyst yield was achieved at pH 6 (15). Finally, our system did not include a basal lamina substitute, and while S2 cells were found not to be essential for ookinete transformation, they proved necessary for oocyst maintenance *in vitro*, in agreement with what has previously been reported (29).

Luciferase-expressing P. berghei parasites have already been described as a valuable tool to assess hepatic infection *in vitro* and *in vivo* (30, 31), as well as to measure blood parasitemia in rodent models of infection (32). Here, a firefly luciferase-expressing P. berghei parasite line was employed to monitor the parasite's sporogonic development *in vitro*, as well as a tool for the identification of compounds with TB activity. Our assay might potentially be improved in the future by the use of the recently reported P. berghei line expressing the novel luciferase enzyme NanoLuc (PbNLuc), which has been shown to deliver a significantly enhanced luminescence signal, enabling single parasite detection in various stages of its life cycle, including in the mosquito vector (33). Another possible improvement of the assay might come from the use of a second reporter gene, placed under the control of a sporozoite-specific promoter, which would enable the specific monitoring of sporozoite formation and maturation.

Our *in vitro* study identified several sporontocidal compounds with potential TB activity. Unlike gametocytocidal compounds, sporontocidal drugs should ideally have a long half-life to interrupt transmission, as gametocytes can circulate for long periods of time before being ingested by a mosquito. However, sporontocidal agents may also present advantages over gametocytocidal compounds, as the former can interrupt transmission by mosquitoes taking a postinfection blood meal. In fact, the *in vivo* activity of compounds identified in our *in vitro* assay was subsequently confirmed for Cy, Th, and At by adding these compounds to infected mosquito sugar feeds as previously described (34). Besides substantial activity against P. falciparum blood stages (35), Cy has been shown to inhibit P. falciparum exflagellation and P. berghei ookinete formation (9), appearing as one of the compounds with the most notable activity in a comparative study of potential TB compounds (9). Since Cy is a known inhibitor of translation (36), its inhibitory activity on exflagellation is consistent with the notion that protein synthesis is an important component of the morphological changes that occur upon activation of the exflagellation process (37). In our assay, Th displayed the broadest spectrum of activity and presented the strongest sporontocidal effect. Despite a very promising biological profile, Th has not been developed for clinical use, primarily because of its low aqueous solubility and formulation issues (38). However, given their dual activity on two independent targets, the parasite proteasome and the apicoplast, with the capacity to eliminate both intraerythrocytic asexual and transmission stages of the parasite, Th derivatives represent promising candidates for malaria therapy (39). At also demonstrated strong activity against ookinete formation and oocyst maturation in our study. Of note, hepatic infection of naive mice was completely abrogated following the administration of At to infected mosquitoes. Interestingly, At in serum collected from human volunteers was previously shown to block P. berghei development from ookinete to oocyst (40). This compound is frequently paired with other agents in patients with malaria. When administered alone, it is associated with unacceptable recrudescence rates and decreased parasite susceptibility following treatment (41). However, given its TB potential, an investment in new synergetic partners for At may be of interest.

One of the first steps toward finding TB compounds is to screen commercially available antimalarials with gametocytocidal activity (42). Although standard membrane feeding assays (SMFAs) are considered the gold standard for evaluating the infectivity of compound-treated gametocytes (43), this type of assay requires dissection and microscopic evaluation of individual mosquitos, which are very labor intensive. Assays measuring the viability or development of gametocytes without the need for mosquito dissection are comparatively inexpensive and highly scalable and will therefore continue to be required as a prioritization step in the screening pipeline for TB compounds (44). Although the development of GFP-luciferase-expressing parasites has allowed for scalability of SMFAs (45), it is still important to establish filtering assays with higher throughput to prioritize compounds for subsequent evaluation in SMFAs. Even with *in vitro* culture, where protocols for *in vitro* generation of P. falciparum ookinetes have been made available, the throughput of such assays remains a concern (46, 47). In a recent comparison of SMFAs and alternative assays for identification of compounds with P. falciparum transmission-reducing activity, a luciferase-based assay was shown to yield the strongest agreement with SMFAs (44). Thus, our assay appears as a valid tool for *in vitro* screening of TB compounds acting at various stages of the parasite's sporogonic development for subsequent *in vivo* evaluation. Furthermore, in facilities were the availability of *in vivo* models is sparse, this assay represents a relatively inexpensive alternative for compound screening. It establishes a fast method for screening a wide range of compounds in a relatively short time, allowing the specific assessment of compound activity against the various stages of Plasmodium sporogonic development.

## MATERIALS AND METHODS

### Experimental animals and P. berghei ANKA reference line.

Female Swiss OF1 mice (6 to 8 weeks old) from Charles River were used. The cloned reference line cl15cy1 of the ANKA isolate of P. berghei (48) was used to obtain the transgenic P. berghei line that expresses the *gfp-luc* fusion gene under the control of the *csp* promoter (PBANKA_0403200) integrated into the silent *230p* gene locus (PBANKA_0306000).

### Generation and characterization of P. berghei *csp-gfp-luc*-expressing line.

The *csp* promoter was PCR amplified with primers 2590 (5′ CCGGATATCACATAAAAGGGAATATGGAATATACTAGC) and 2591 (5′ CGCGGATCCAAATATATGCGTGTATATATAGATTTTG) and cloned as a BamHI/EcoRV fragment into plasmid pL1141 (exchanging the *ama1* promoter for the *csp* promoter) to create pCSGFP203p. Then the NcoI/ScaI fragment of pL1156 was introduced to create pL1161.

The final DNA construct was linearized with SacII before transfection. Transfection, selection, and cloning of transformed P. berghei parasites were performed by using standard genetic modification techniques (48) with P. berghei ANKA cl15cy1 as the parent parasite line. Cloned parasite lines (see Fig. S1; exp. 784; PbCSPGFP-Luc) were obtained by the limiting dilution method. Correct integration of DNA constructs and disruption of genes were verified by Southern analyses of pulsed-field gel (PFG)-separated chromosomes (48). PFG-separated chromosomes were hybridized with the 3′ untranslated region of the *P. berghei dhfr/ts* gene recognizing the endogenous *dhfr/ts* locus on chromosome 7 and the *GFP-Luc-cs* expression cassette on chromosome 3.

### Ookinete production.

P. berghei ANKA expressing GFP and luciferase under the control of the P. berghei circumsporozoite protein (*Pb*CSP) promoter (line 784cl1, RMgm-152, *Pb*CSPGFP-Luc) was maintained in A. stephensi mosquitoes and BALBc/byJ mice. To maintain gametocyte infectivity, only up to six passages of parasites from infected mice to naive mice were performed. Briefly, BALBc/byJ mice were treated with 0.1 ml of phenylhydrazine (25 mg/ml) 3 days prior to infection with 1 × 10^7^ P. berghei-infected red blood cells (iRBCs) obtained from a donor mouse, which was infected either from a previously infected mouse or from a frozen vial of iRBCs. Parasitemia and gametocytemia were monitored by the analysis of Giemsa-stained tail blood smears, and the presence of exflagellating gametocytes in ookinete medium (1:4 dilution) was monitored. Three days after infection, blood collected by heart puncture from two mice was pooled and washed with RPMI at 37°C, followed by centrifugation at 1,100 × *g* for 10 min at 37°C. After washing, 5, 10, or 15 μl of blood containing exflagellating gametocytes was mixed with medium supplemented for ookinete formation (RPMI 1640 [Sigma], 25 mM HEPES, 0.4 mM hypoxanthine, 100 mM xanthurenic acid [Fluka 85570], 10% [vol/vol] FBS, pH 7.6) in a final volume of 200 μl and cultured in 96-well plates for 24 h at 19°C. Additionally, blood containing exflagellating gametocytes was mixed with the ookinete medium in a 1:20 ratio and cultured in T75 flasks for 22 to 24 h at 19°C. Following incubation, ookinete enrichment was performed as previously described (9), with some modifications. Briefly, cultured blood was collected and erythrocytes were lysed for 15 min on ice with 30 volumes of ice-cold 0.17 M ammonium chloride. Lysed erythrocytes were removed by washing with RPMI, and ookinetes were purified by centrifugation on a 63% Nycodenz cushion at 650 × *g* at 4°C for 30 min. Following centrifugation, the ookinete-containing interface was collected, washed in ice-cold RPMI, and resuspended in 0.5 to 1 ml of oocyst medium (see paragraph on oocyst cultures below). Ookinete conversion rates were determined by counting all Pbs21-positive cells 22 to 24 h after *in vitro* incubation of infected blood in ookinete culture medium after staining with 3.3 μg/ml antibody (monoclonal antibody 13.1) for 2 h. After washing, samples were further incubated with 6.66 μg/ml Alexa Fluor 488 (Invitrogen)-conjugated anti-rabbit IgG for 1 h. The ookinete conversion rate is the percentage of ookinetes (mature and retort forms) of the total number of Pbs21-positive cells (ookinetes and unfertilized gametes).

### Oocyst cultures.

Purified ookinetes were seeded with Drosophila melanogaster S2 cells (Drosophila Genomics Resource Center, Bloomington, IN) at a 1:10 ratio (1 × 10^4^ ookinetes and 1 × 10^5^ S2 cells) in Schneider's medium (Sigma S0146) supplemented with 15% FBS, penicillin-streptomycin (50 U/ml and 50 μg/ml, respectively), and gentamicin (50 μg/ml) to promote oocyst development. Oocysts were cocultured with D. melanogaster S2 cells in flat-bottom 96-well plates (Corning) for 21 days at 19°C. One-quarter of the medium was changed three times a week (every 48 to 72 h), and 1 × 10^5^ S2 cells were added once or twice a week. In parallel, S2 cells were maintained at 27°C in Schneider's medium (Sigma S0146) supplemented with 10% FBS and penicillin-streptomycin (50 U/ml and 50 μg/ml, respectively).

### Bioluminescence assay.

A bioluminescence assay was used to assess the development of the mosquito stages of the parasite. To monitor the development of gametocytes into ookinetes, firefly luciferase-expressing parasites (*Pb*CSPGFP-Luc) were collected from the ookinete culture at five time points over a period of 24 h. Parasites were then collected from oocyst cultures every day for 21 days of culture to assess the dynamics of firefly luciferase expression during oocyst development. On the basis of the results obtained, in subsequent experiments performed to evaluate the effect of compounds on the development of different Plasmodium mosquito stages, parasites were collected at three different time points (see paragraph on evaluation of antiplasmodial compounds *in vivo* below). The bioluminescence assay was performed with the Firefly Luciferase Assay kit (Biotium) in accordance with the manufacturer's instructions, with some modifications. Briefly, the whole well contents were collected, washed with phosphate-buffered saline (PBS), frozen in 75 μl of lysis buffer, and stored at −20°C until further use. Collected samples were lysed, and 30 μl of the resulting supernatant was transferred into white 96-well plates. Fifty microliters of d-luciferin in firefly luciferase assay buffer (1:50 ratio) was added to the samples, and the parasite load was determined by measuring luminescence intensity with a microplate reader (Infinite M200).

### Live imaging of oocyst cultures.

Live imaging of *Pb*CSPGFP-Luc oocyst cultures was performed in black 96-well plates (Corning) on either a Zeiss Axiovert 200M or a Zeiss Cell Observer WF fluorescence microscope with a 40× objective. Oocyst development and sporulation were monitored on days 3, 5, 8, 10, 15, 18, and 21 of culture. For quantification of oocyst numbers and areas, 50 images per well were acquired and analyzed with the Fiji software (49).

### Immunofluorescence microscopy.

Blood was collected from mice infected with *Pb*CSPGFP-Luc 3 days after infection as described above. Fifty microliters of the blood containing gametocytes was centrifuged immediately, while the remainder was centrifuged after incubation for 24 h in the medium to promote ookinete growth. After centrifugation at 4,000 rpm for 5 min, samples were first fixed with 4% paraformaldehyde (PFA) and 0.0075% glutaraldehyde in PBS for 30 min, permeabilized by incubation with 0.1% Triton X-100–PBS for 10 min, and then blocked with 3% bovine serum albumin (BSA)–PBS for 1 h. To characterize the expression of the Pbs21 protein, gametocytes were first incubated with 3.3 μg/ml 13.1 anti-Pbs21 primary antibody (kindly provided by Jorge Santos) for 2 h. After washing, samples were further incubated with 6.66 μg/ml Alexa Fluor 555 (Invitrogen)-conjugated anti-mouse IgG, 6.66 μg/ml Alexa Fluor 488 (Invitrogen)-conjugated anti-GFP tag antibody, and 5 μg/ml Hoechst for 1 h. To characterize CSP expression, ookinetes of *Pb*CSPGFP-Luc and *Pb*GFP-Luc_con_ were incubated with 5 μg/ml anti-CSP 3D11 antibody (50) for 2 h and then incubated with 6.66 μg/ml Alexa Fluor 555 (Invitrogen)-conjugated anti-mouse IgG, 6.66 μg/ml Alexa Fluor 488 (Invitrogen)-conjugated anti-GFP tag antibody, and 5 μg/ml Hoechst for 1 h. All antibodies were diluted in 3% BSA–PBS, and all steps were performed at room temperature. Finally, samples were mounted with Fluoromount-G (SouthernBiotech) on polylysine coverslips (Corning) on microscope slides and images were acquired on Zeiss LSM 880 and Zeiss LSM 710 fluorescence microscopes. To characterize CSP expression and to image parasite nuclei, oocysts were cultured in eight-well chambers, air dried overnight, fixed for 10 min with 4% PFA–PBS, and permeabilized and blocked for 1 h with 0.1% Triton X-100–1% BSA–PBS. Samples were then incubated with 10 μg/ml anti-CSP 3D11 antibody (50) for 1 h and subsequently with 4 μg/ml Alexa Fluor 594 (Invitrogen)-conjugated goat anti-mouse IgG secondary antibody for 1 h. All incubations were performed at room temperature, and all washing steps were performed with 0.05% Triton X-100–1% BSA–PBS, which was also used to prepare antibody dilutions. Nuclei were stained with 5 μg/ml Hoechst 33342–PBS. Samples were mounted in Fluoromount-G (SouthernBiotech 0100-01), and images were acquired on either a Zeiss LSM 880 or a Zeiss LSM 710 fluorescence microscope and processed with the Fiji software (49).

To characterize CSP expression *in vivo*, BALB/cJ (Charles River) mice were infected by intraperitoneal injection of 1 × 10^7^ *Pb*CSPGFP-Luc and *Pb*GFP-Luc_con_-iRBCs. Parasitemia was monitored by analysis of Giemsa-stained tail blood smears, and when it reached around 3% and after the confirmation of male gametocyte exflagellation, these mice were used to infect A. stephensi mosquitoes raised in the Instituto de Medicina Molecular (IMM) insectary facility. Following their blood meal, mosquitoes were starved and kept at 20°C and 80% humidity under a 12-h light-dark cycle for 24 h. After 24 h, approximately 20 midguts per parasite strain were collected, fixed with 4% PFA for 1 min, and then washed in PBS. Midguts were opened longitudinally, and the contents were removed in PBS, fixed for 30 min at room temperature, and permeabilized and blocked for 1 h with 0.1% Triton X-100–1% BSA–PBS. Samples were then incubated with 5 μg/ml anti-CSP 3D11 antibody and 6.6 μg/ml 13.1 anti-Pbs21 antibody overnight at 4°C and subsequently with 6.66 μg/ml Alexa Fluor 594 (Invitrogen)-conjugated goat anti-mouse IgG secondary antibody and 6.66 μg/ml Alexa Fluor 488 (Invitrogen)-conjugated IgG secondary antibody. Midguts were mounted on a microscope slide with Fluoromount-G (SouthernBiotech 0100-01), and images were acquired with a Zeiss LSM 880 fluorescence microscope and analyzed with the Fiji software.

### Western blot analyses.

To evaluate *Pb*CSP expression throughout the transformation of parasites from gametocytes to ookinetes *in vitro*, cultured blood of ookinete cultures of *Pb*CSPGFP-Luc parasites produced as described above in 96-well plates was collected at 0, 6, 12, 18, and 24 h postincubation. After collection, samples were lysed with firefly luciferase lysis buffer from the Firefly Luciferase Assay kit (Biotium) in accordance with the manufacturer's instructions. Samples were prepared by adding 4× Laemmli sample buffer (diluted in β-mercaptoethanol) to the lysed parasite solution (1:3) and boiled at 95°C for 5 min. Lysates were then separated by gel electrophoresis on a precast Mini-PROTEAN TGX gel (Any Kd; Bio-Rad), and lysates of 1,000 *Pb*CSPGFP-Luc sporozoites dissected in lysis buffer (25 mM Tris-HCl [pH 7.4], 1% Triton X-100, 10% glycerol) with protease inhibitor (1:50) were used as a positive control. The approximate molecular weights of the proteins were deduced by comparison with a prestained protein ladder (Bio-Rad). The gel was transferred to a nitrocellulose membrane with iBlot (Invitrogen). Membranes were blocked for 30 min in PBS–0.1% (vol/vol) Tween–5% (wt/vol) milk, and then incubated with anti-CSP 3D11 antibody (1:1,000) overnight at 4^a^C. Membranes were washed five times for 5 min in PBS–0.1% (vol/vol) Tween and then incubated for 1 h with horseradish peroxidase-conjugated anti-mouse IgG antibody (1:5,000) and washed as described above. Membranes were developed with the Immobilon Western blot kit (Millipore) and developed with the ChemiDoc XRS+ chemiluminescence detection system (Bio-Rad). The blots were analyzed with Image Lab software (Bio-Rad).

To confirm CSP expression in ookinete samples produced *in vitro*, purified ookinete samples of *Pb*CSGFP-Luc and *Pb*GFP-Luc_con_ were collected at 24 and 48 h postincubation and lysed has described above. Samples were treated with Benzonase and MgCl_2_ (1:100 dilution) to degrade nucleic acids, In parallel, midguts of A. stephensi mosquitoes infected with both parasite strains as previously described were collected at 24 and 48 hpi and lysed in lysis buffer (25 mM Tris-HCl [pH 7.4], 1% [vol/vol] Triton X-100, 10% [vol/vol] glycerol) with protease inhibitor (1:50). Samples were prepared by adding 4× Laemmli sample buffer (diluted in β-mercaptoethanol) to the lysed parasite solution (1:3); boiled at 95°C for 5 min; and separated, transferred, and developed as described above.

### Evaluation of antiplasmodial compound activity *in vitro*.

The effects of Az, At, Ch, Cy DA, Ha, Lu, Py, Po, and Th on Plasmodium mosquito stages were evaluated. The compounds were dissolved in dimethyl sulfoxide (DMSO), and the amount of DMSO equivalent to that present in the highest compound concentration was used as a control. To assess the effects of the compounds on the ookinetes, a 10 μM concentration of each compound was added to blood containing mature gametocytes after 1 h of incubation to evaluate their effect on ookinete formation and development when the zygote is already formed. To determine the intensity of the bioluminescence signal, parasites were collected from the ookinete cultures after 24 h of incubation. To assess the effects of the compounds on oocyst development, the compounds were mixed with mature ookinetes or added to oocyst cultures after 3 days of culture. Az, Ch, DA, Ha, Lu, Py, Po, Th, At, and Cy were assayed at 10 μM. Compounds were replenished when the medium was changed to maintain the appropriate final concentration. When the compounds were mixed with the ookinetes, parasites were collected after 3 days of culture to assess the effects of the compounds on the early stages of oocyst development. When the compounds were added after 3 days of oocyst culture, parasites were collected after 15 days of oocyst culture to evaluate the compounds' effects on oocyst development when added to already formed young oocysts. IC_50_s for oocyst growth were calculated for Cy (assayed at 0.005, 0.025, 0.05, 0.5, 1, 5, and 10 μM), Py, and Th (assayed at 0.05, 0.5, 1, 5, 10, 25, and 50 μM). IC_50_s were estimated by nonlinear regression analysis with the sigmoidal dose-response equation in GraphPad Prism (version 5.00; GraphPad Software, La Jolla, CA).

### Evaluation of antiplasmodial compounds *in vivo*.

To assess the *in vivo* activities of compounds previously identified *in vitro*, BALB/cJ mice (Charles River) were infected by intraperitoneal injection of 10^7^ *Pb*GFP-Luc_con_-iRBCs. Parasitemia was monitored by the analysis of Giemsa-stained tail blood smears, and when it reached around 3% and after the confirmation of male gametocyte exflagellation, these mice were used to infect A. stephensi mosquitoes raised in the IMM insectary facility. Following their blood meal, mosquitoes were starved and kept at 20°C and 80% humidity under a 12-h light-dark cycle for 2 days. A solution of 100 mg/ml glucose and 2 mg/ml *p*-aminobenzoic acid mixed with selected compounds, At, Cy, DA, and Th, at a 50 μM concentration was provided to mosquitoes for 8 days in 384-well plates and replaced every 2 days. On day 10 after mosquito infection, approximately 30 midguts per experimental condition were collected and fixed with 4% PFA for 20 min. To determine oocyst numbers and development, midguts were mounted on a microscope slide with Fluoromount-G (SouthernBiotech 0100-01) and images were acquired with a Leica DM5000B and analyzed with Fiji software (49). To assess *in vivo* hepatic infection, the remaining mosquitoes were kept under standard diet conditions and on day 21 of infection, approximately 20 mosquitoes were allowed to feed on naive BALB/cJ mice (Charles River) for 30 min. On the following day, salivary glands were dissected and salivary gland sporozoite numbers were determined.

### Quantification of *in vivo* hepatic infection.

Liver parasite burdens of infected mice were quantified by qRT-PCR as previously described (51). Briefly, livers were collected at 46 h postinfection and immediately homogenized in a denaturing solution (4 M guanidine thiocyanate, 25 mM sodium citrate [pH 7.0], 0.5% sarcosyl, and 0.7% β-mercaptoethanol in diethyl pyrocarbonate-treated water). Total RNA was extracted with the TripleXtractor directRNA kit (GRiSP) in accordance with the manufacturer's protocol. One microgram of total RNA was converted to cDNA (NZY First-Strand cDNA synthesis kit, no oligonucleotides, [NZYTech]), and parasite loads were quantified by qRT-PCR with primers specific for the P. berghei 18S rRNA (5′-AAGCATTAAATAAAGCGAATACATCCTTAC-3′ and 5′-GGAGATTGGTTTTGACGTTTATGTG-3′). Primers for the well-established housekeeping gene for hypoxanthine-guanine phosphoribosyltransferase (5′-TTTGCTGACCTGCTGGATTAC-3′ and 5′-CAAGACATTCTTTCCAGTTAAAGTTG-3′) were used for normalization of infection loads in all experiments. The qRT-PCRs were performed in a total volume of 20 μl in a ABI Prism 7500 Fast system (Applied Biosystems) with the iTaq Universal SYBR green kit (Bio-Rad) as follows: 50°C for 2 min, 95°C for 10 min, 40 cycles of 95°C for 15 s and 60°C for 1 min, a melting stage of 95°C for 15 s, 60°C for 1 min, and 95°C for 30 s. The delta-delta cycle threshold (ΔΔ*C_T_*) relative quantification method was used for analysis of qRT-PCR results.

### Statistical analysis.

Statistically significant differences between control and treated conditions were analyzed with the Mann-Whitney nonparametric test by using a 95% confidence interval. Differences were considered not be significant at a *P* value of >0.05. Under this value, all differences were considered to be statistically significant. All statistical tests were performed by GraphPad Prism (version 5.00; GraphPad Software, La Jolla, CA).

### Ethics statement.

All work with laboratory animals was performed in accordance with national and European regulations. All protocols were approved by the animal experimentation ethics committee of the Instituto de Medicina Molecular. All animal experiments performed at the Leiden University Medical Center were approved by the Animal Experiments Committee of the Leiden University Medical Center (DEC 12042, DEC 12043). The Dutch Experiments on Animal Act was established under European guidelines (EU directive no. 86/609/EEC regarding the protection of animals used for experimental and other scientific purposes).

### Accession number(s).

Details of the DNA construct used in this study have been submitted to the database of genetically modified rodent malaria parasites (RMgmDB, http://www.pberghei.eu/; ID RMgm-152).

## Supplementary Material

Supplemental material
