# Supplementary material for: Bioluminescence Method for In Vitro Screening of Plasmodium Transmission-Blocking Compounds
Source: Antimicrob Agents Chemother. 2017 May 24;61(6):e02699-16. doi: 10.1128/AAC.02699-16 (PMC5444155; doi:10.1128/AAC.02699-16)
Supplement: Supplemental material [file supp_61_6_e02699-16__index.html]

Bioluminescence Method for In Vitro Screening of Plasmodium Transmission-Blocking Compounds — Supplemental material 

# Bioluminescence Method for *In Vitro* Screening of Plasmodium Transmission-Blocking Compounds

## Supplemental material

- Supplemental file 1 -

  Fig. S1-S4

  PDF, 1.8M
