# Supplementary material for: Bioluminescence Method for In Vitro Screening of Plasmodium Transmission-Blocking Compounds
Source: Antimicrob Agents Chemother. 2017 May 24;61(6):e02699-16. doi: 10.1128/AAC.02699-16 (PMC5444155; doi:10.1128/AAC.02699-16)
Supplement: Supplemental material [file AAC.02699-16_zac006176219s1.pdf]

A bioluminescence method for *in vitro* screening of *Plasmodium* transmission-blocking compounds

Raquel Azevedo, Marija Markovic, Marta Machado, Blandine Franke-Fayard, António M. Mendes, Miguel Prudêncio

Supplemental Material (4 pages)

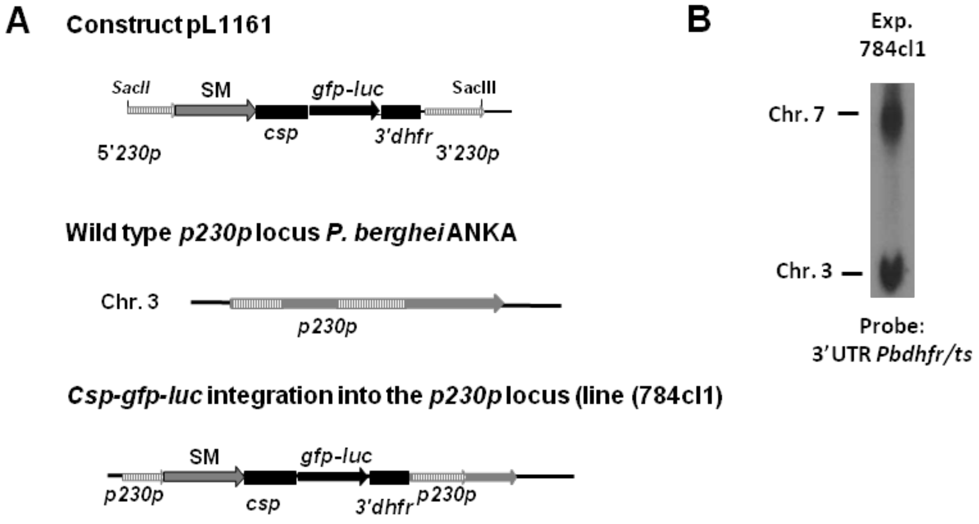

FIG. S1. Generation and genotyping of parasites expressing GFP-luciferase under control of the csp promoter (*Pb*CSPGFP-Luc; line 784cl1)

A. Schematic representation of the plasmid, the wild-type gene *230p* locus before and after incorporation of the construct pL1161 used to generate the transgenic line expressing GFP-luciferase under control of the *csp* promoter (exp 784). The construct contains the *Toxoplasma gondii dihydrofolate reductase-thymidylate synthase (dhfr-ts)* selectable marker cassette (SM: grey arrow) and the *csp-GFP-Luc* expression cassette (black arrow) and integrates by double cross-over homologous recombination into the *230p* locus. B. Diagnostic Southern analysis of pulsed field gel-separated chromosomes confirms correct integration of the csp-GFP-Luc expression cassette in cloned line 784cl1. Separated chromosomes were hybridized with the 3'UTR of *P. berghei dhfr/ts* gene recognizing the endogeneous *dhfr/ts* locus on chromosome 7 and the *csp-GFP-Luc* cassette on chromosome 3.

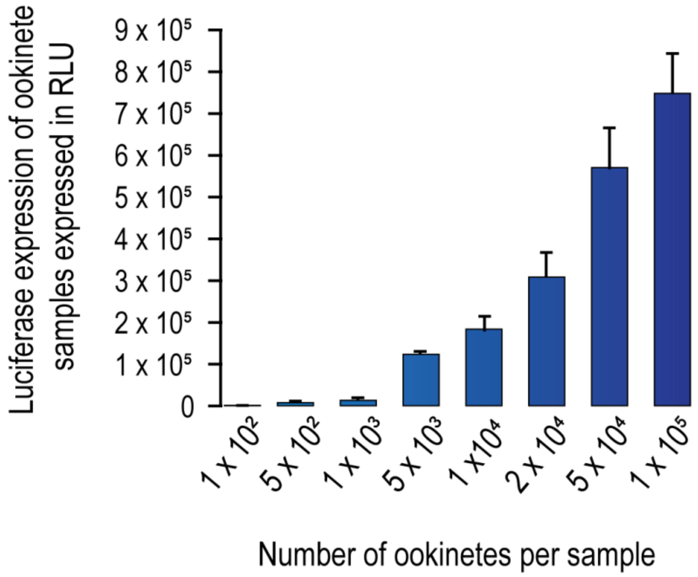

FIG. S2. Luciferase expression of ookinete samples.
Bioluminescence of serial dilutions of samples of purified ookinetes produced *in vitro*. Data is expressed as a mean RLU +/- SD (triplicate wells).

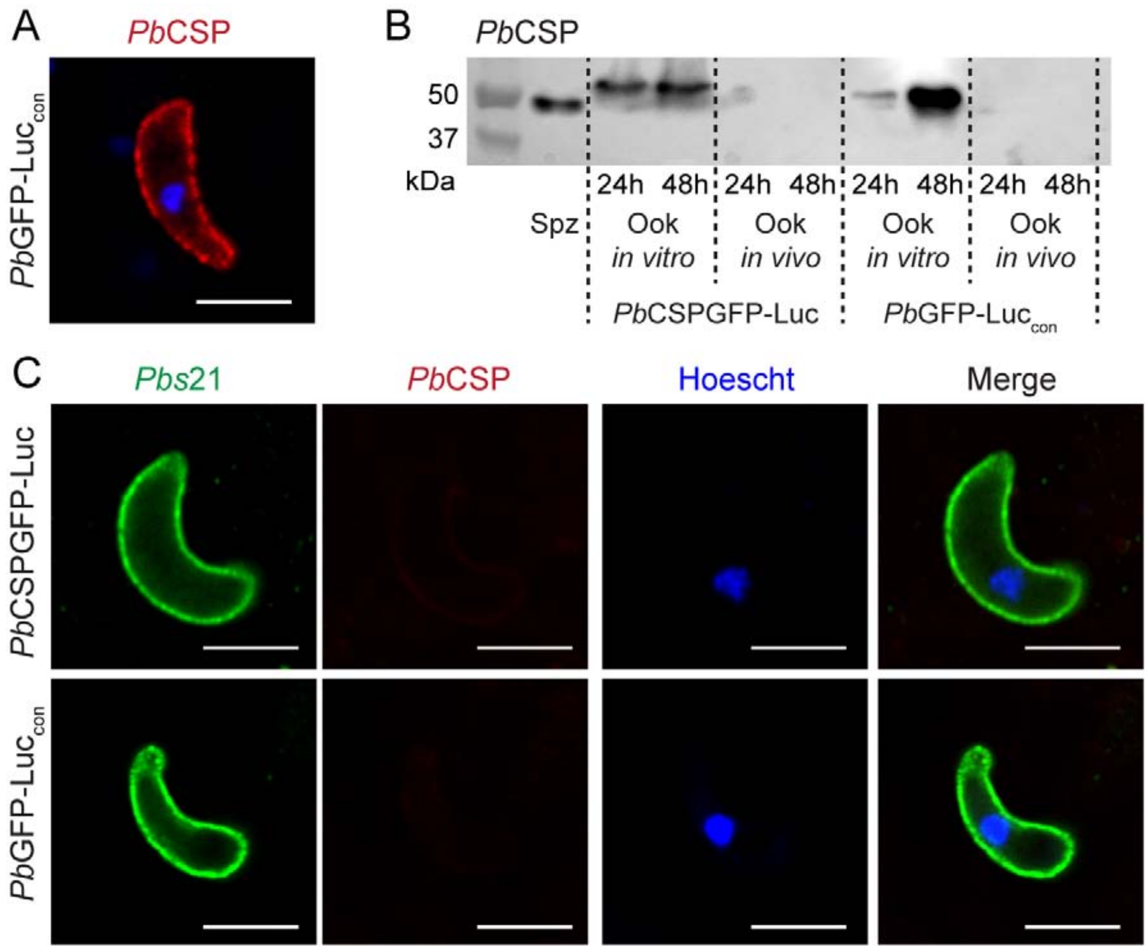

FIG. S3. CSP expression in ookinetes *in vitro* and *in vivo*.

A. Immunofluorescence staining of *Pb*GFP-Luc$_{Con}$ ookinetes (red: *Pb*CSP, blue: nuclei). Scale bar: 5 µM. B. Western blot analysis of *Pb*CSP in samples from *Pb*CSPGFP-Luc and *Pb*GFP-Luc$_{Con}$ parasites, collected either *in vitro* or *in vivo* 24 and 48 hours after the start of the culture or of mosquito infection, respectively. C. Immunofluorescence microscopy analysis of *Pb*CSPGFP-Luc and *Pb*GFP-Luc$_{Con}$ parasites collected *in vivo* 24 hours after mosquito infection.

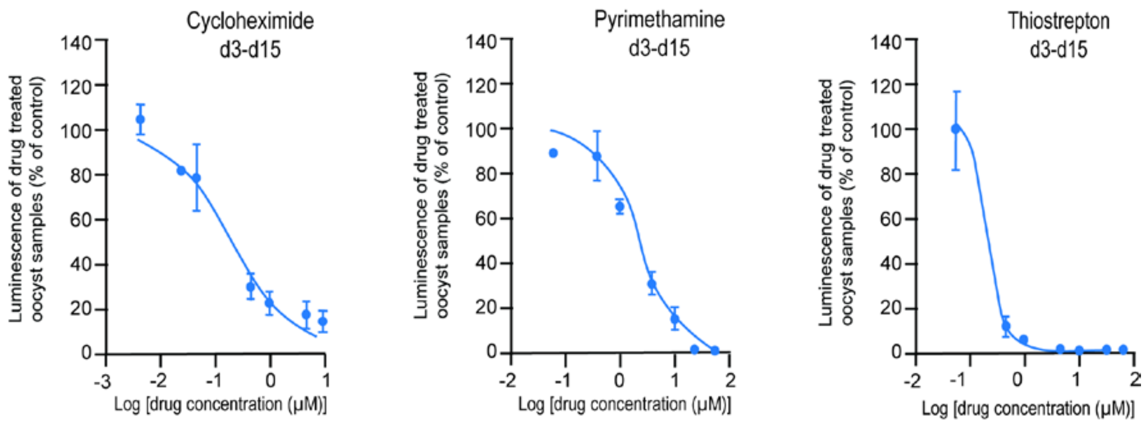

FIG. S4. IC50 determination for selected compounds active against oocyst maturation.

IC_{50} values for cycloheximide, pyrimethamine and thiostrepton were 0.28 ± 0.32 µM, 1.93 ± 1.72 µM and 1.16 ± 0.10 µM, respectively.
